# The Effect of Gestational Diabetes on Neonatal Outcomes in Jeddah City: A Retrospective Study

**DOI:** 10.1002/hsr2.71858

**Published:** 2026-03-04

**Authors:** Ahmad Ismail, Ezzi Elham Mohammed Ahmed, Al‐Shraifeen Ali Ahmad, Ahmed Ragab, Mohammad Othman

**Affiliations:** ^1^ Nursing Department, Fakeeh College for Medical Sciences Fakeeh Care Group Jeddah Saudi Arabia; ^2^ Nursing Department, Neonatal Intensive Care Nursing Master Program, Fakeeh College for Medical Sciences Fakeeh Care group Jeddah Saudi Arabia; ^3^ Clinical Sciences Department‐MBBS Program, Fakeeh College for Medical Sciences Fakeeh Care Group Jeddah Saudi Arabia

## Abstract

**Background and Aims:**

The prevalence of gestational diabetes mellitus (GDM) in Saudi Arabia is believed to affect 36% of all pregnant women. Many adverse outcomes affect the lives of the neonates of these mothers. Therefore, this study aims to assess the association between GDM and neonatal health outcomes in Jeddah City, Saudi Arabia.

**Methods:**

A retrospective cohort design was used. Two hundred neonatal records were reviewed: 100 for neonates of mothers who had GDM and 100 without GDM for control. The two groups were compared in terms of neonatal outcomes.

**Results:**

The gestational age and the glycemic level of neonates of mothers without GDM were significantly higher than those with GDM (39± weeks vs. 37 weeks and 63± vs. 55 mg/dL, *p* ≤ 0.05). Neonates of mothers with GDM required cesarean section deliveries more than neonates of mothers without GDM (92% vs. 40%, *p* ≤ 0.05). Also, these neonates were more prone to develop respiratory distress syndrome and congenital anomalies than neonates of mothers without GDM (21% vs. 11% and 17% vs. 4%, *p* ≤ 0.05).

**Conclusion:**

This study provides empirical evidence demonstrating the negative effect of GDM on neonatal health outcomes, mainly in terms of hypoglycemia, respiratory distress syndrome, and congenital anomalies. Therefore, it highlights the importance of adequate glycemic control by specifying proper treatment together with dietary intervention and exercise programs to improve outcomes. All authors have read and approved the final version of the manuscript. Corresponding author had full access to all the data in this study and takes complete responsibility for the integrity of the data and the accuracy of the data analysis.

## 1 Introduction

Gestational diabetes mellitus (GDM) is a glucose intolerance that begins or is first identified during pregnancy [1, 2]. The global prevalence of GDM is 14%. The regional prevalence in North America and the Caribbean is 7.1%, in Europe 7.8%, in South America and Central America 10.4%, in Africa 14.2%, in the Western Pacific 14.7%, in South‐East Asia 20.8%, and the highest is 27.6% in the Middle East and North Africa [3, 4].

In Saudi Arabia, the prevalence of GDM is even higher than the global prevalence [5]. The prevalence of GDM in Saudi women was reported in 2010 as 12.5% [6]. In 2014, it was reported to be higher (36.6%) when applying the International Association of Diabetes and Pregnancy Study Groups (IADPSG) criteria for diagnosis [7]. In 2015, a study reported an increased prevalence of GDM in Saudi Arabia (51%) when applying the IADPSG criteria [8]. A more recent study reported a higher prevalence of GDM (55%) [9]. These values regarding the prevalence of GDM in Saudi Arabia are alarming [10].

Gestational diabetes mellitus can lead to short and long‐term consequences for both the mothers and their babies. Newborns of women diagnosed with GDM have high risks of developing many adverse outcomes, such as large for gestational age (LGA) and preterm births, compared to newborns of women without GDM [11]; Muche, Olayemi, & Gete, 2020 [12, 13]. It has been also shown that it is commonly associated with low (APGAR score “Appearance, Pulse, Grimace, Activity and Respiration Score”), more neonatal intensive care unit (NICU) admission, hyperbilirubinemia, congenital malformations, respiratory distress syndrome (RDS), hypoglycemia, and macrosomia [2, 12, 14]. Diabetes during pregnancy is also associated with some long‐term effects, such as an increased risk of obesity and diabetes development in both mothers and children [15]. However, neonates of women with well‐controlled diabetes are healthier than those of women with uncontrolled diabetes during pregnancy [5, 16].

In Saudi Arabia, several studies were conducted on the prevalence of GDM and the maternity and neonatal outcomes [5, 8, 9, 17]. To date, no study has been identified that assesses the adverse neonatal outcomes associated with GDM in Jeddah City. Therefore, this study was conducted aiming to assess the association between GDM and neonatal health outcomes by comparing two equal groups of mothers who were diagnosed with GDM versus those who were not.

## 2 Material and Methods

### 2.1 Study Design

This study used a retrospective cohort study design. Newborn outcomes of women diagnosed with GDM were compared with the neonatal outcomes of women without GDM in Saudi Arabia at a private hospital by reviewing their records.

### 2.2 Study Setting

This study was conducted at a private hospital in Jeddah, Saudi Arabia. This hospital handles approximately 3000 deliveries each year. The hospital is occupied with 38 incubators/beds dedicated to Neonatal Intensive Care Unit (NICU) care. The medical facility contributes to health education and teaching through bedside care and enhances disease treatments by conducting medical research and training.

### 2.3 Study Population

The study population included the records of all pregnant women diagnosed with GDM who gave birth at a private hospital in Jeddah, Saudi Arabia and their neonates from January 2022 to December 2022, giving a total of 3000 births per year.

### 2.4 Sample and Sampling

A non‐probability purposive sampling strategy was used. Pregnant women diagnosed solely with GDM were intentionally targeted, along with a comparable group of pregnant women without GDM. The sample size was calculated based on the effect size. The main test used in this study to compare the two groups of neonates (neonates born to mothers with GDM and neonates born to mothers without GDM) was the independent *t*‐test. The effect size of the independent *t*‐test is Cohen's *d*. Since no similar study compared the same variables and used the same statistical test, we assumed a moderate effect size (0.5). Using the power analysis software (G*Power), the minimum required sample size on a power level of 80% and P of 0.05 was 128 (64 per group). In this study, we recruited 200 participants (100 per group). This would give more accurate inferences about the study population, reduce sampling error, increase power, and reduce the risk of type II errors.

### 2.5 Inclusion Criteria and Exclusion Criteria

The current study included (1) records of pregnant women diagnosed with GDM and (2) women who gave birth (alive) to a neonate at one private hospital in Jeddah, Saudi Arabia. In addition, records of women without diabetes were also accessed to obtain the required sample. Their inclusion criteria were: (1) women who gave birth (alive) to a neonate and (2) had no complications after giving birth. Women with pre‐pregnancy diabetes mellitus, multiple pregnancy, incomplete patient records, and women with other comorbid conditions were excluded from the study since having another illness may influence health outcomes.

### 2.6 Data Collection Tools

The Data was collected using two validated checklists. These checklists were developed based on previous literature. The first one contained maternal information such as age, Body Mass Index (BMI), gravidity, parity, and Hemoglobin A1c to assess the average blood glucose level in the third trimester. The second checklist was used to assess the neonatal health outcomes. It included mode of delivery, baby gender (male, female), gestational age at birth (by weeks), baby's birth weight, Apgar score, glycemic level if the baby needed newborn resuscitation (basic or advanced), admission to the NICU, and if the baby had respiratory distress syndrome or congenital abnormalities. In our study, neonatal blood glucose levels were obtained from the neonates' electronic medical records, where measurements were performed as part of routine hospital protocol for newborns at risk (including infants of mothers with GDM). The timing of the first glucose measurement was within the first hour after birth, with subsequent measurements taken at 3 h of life and then every 3–6 h as clinically indicated until stable glucose levels were achieved. The diagnosis of congenital anomalies was based on the documented assessments in the neonates' electronic medical records. These diagnoses were made by neonatologists and pediatric subspecialists using standard clinical, radiological, and laboratory investigations in accordance with the hospital's neonatal care protocols. The checklists were submitted to experts to check for the categories' face and content validity and relevance. The content validity of the checklist was good (0.80).

### 2.7 Data Collection Procedure

In the current study, the data were collected from patients' electronic records. After obtaining all appropriate ethical approvals, the researcher approached the hospital administration to access the electronic data records. The researcher obtained a password to retrieve the needed data. General screening of patients' records was carried out to identify women diagnosed with GDM and women without GDM. Then their records were accessed to commence data collection using the two checklists designed for this purpose. and was kept in a password‐protected file. Finally, the researcher informed the hospital administration when data collection was completed so that the password to access records would be deactivated.

### 2.8 Data Management and Processing

All data collected were coded and inserted into the Statistical Package for the Social Sciences program (SPSS) version 27.0 (IBM Corp., Armonk, NY, USA). The SPSS was used to analyze the data. Descriptive statistics were calculated for the socio‐demographic data, newborn, and maternal characteristics. Data analyses were used to determine the outcomes associated with the GDM. Independent *t*‐test and chi‐square test were used to compare the neonatal outcomes of women with GDM and women without. These tests were 2‐sided. The significance level was set at ≤ 0.05.

### 2.9 Ethical and Administrative Considerations

Ethical approval was obtained from Fakeeh College for Medical Sciences (460/IRB/2023). Data were kept in a password‐protected file. No one other than the researcher and supervisors could access it. Patients' records were numbered and coded. Patients' names, hospital numbers, and other personal identifiers were not gathered. Data will be kept for 5 years and then will be destroyed using secure deletion.

## 3 Results

A review of 200 pregnancy records (100 with GDM and 100 without) showed that mothers with GDM had a significantly higher mean age (34 vs 30 years), BMI (32 vs 28), gravidity (4 vs 3), and parity (2 vs 1) compared to those without GDM (*p* ≤ 0.05). There was no significant difference between mothers with GDM and those without in the Hemoglobin A1c in the third trimester (*p* = 0.27) (Table 1).

**Table 1:** Characteristics of pregnant mothers diagnosed with GDM versus those not diagnosed with GDM (*n* = 200).

Variable	With GDM, *n* = 100 Mean	Without GDM, *n* = 100 Mean	*t*	*p*	95% CI of the Difference	
					Lower	Upper
Age in years	34.16	29.75	**6.30**	**< 0.001**	**3.03**	**5.80**
Body Mass Index	32.19	28.30	**4.04**	**< 0.001**	**1.98**	**5.79**
Gravidity	3.84	2.75	**4.09**	**< 0.001**	**0.56**	**1.62**
Parity	2.18	1.26	**4.48**	< **0.001**	**0.52**	**1.32**
Hemoglobin A1c in the third trimester	5.50	5.40	1.09	0.27	0.10	0.34

*Note:* Bold values indicate statistically significant.

CI: Confidence Interval.

*t*: Independent *t*‐test.

Table 1: The mean gestational age of neonates of pregnant mothers without GDM was significantly higher than that of those with GDM (39 vs 37, *p* ≤ 0.05). Neonates of pregnant mothers with GDM required cesarean section (CS) for delivery more than neonates of mothers without GDM (92% vs 40%, *p* ≤ 0.05). The glycemic level of neonates of pregnant mothers without GDM was significantly higher than that of mothers with GDM (63 vs 55 mg/dL, *p* ≤ 0.05). Neonates of pregnant mothers with GDM developed RDS more than neonates of mothers without GDM (21% vs 11%, *p* ≤ 0.05). Neonates of pregnant mothers with GDM developed congenital anomalies more than neonates of mothers without GDM (17% vs 4%, *p* ≤ 0.05). The most common types of congenital anomalies in the neonates of mothers with GDM were cardiac (10%) and spina bifida (3%) (Table 2).

**Table 2:** Characteristics of neonates of mothers with GDM and non‐GDM (*n* = 200).

Variable		Neonates of mothers with GDM = 100	Neonates of mothers without GDM = 100	*t*	*p*	95% CI of the Difference	
						Lower	Upper
Gestational Age at Delivery		Mean = 37.32	Mean = 38.54	**4.57**	**< 0.001**	**1.75**	**−0.69**
Birth weight		Mean = 3.10	Mean = 3.06	0.45	0.67	0.13	0.20
APGAR Score/1‐min		Mean = 8.12	Mean = 8.42	1.36	0.16	0.59	0.11
APGAR Score/5 min		Mean = 9.17	Mean = 9.27	0.64	0.53	0.33	0.17
Glycemic Level/mg/dL		Mean = 54.67	Mean = 62.61	**3.07**	**0.002**	**13.13**	**2.74**
		Frequency	Frequency	χ^2^	*p*		
Gender	Male	48	59	2.40	0.12		
	Female	52	41				
Delivery Mode	Normal Vaginal	8	60	**60.25**	**< 0.001**		
	Cesarean Section	92	40				
NICU Admission	Yes	21	13	2.27	0.13		
	No	79	87				
RDS	Yes	21	11	**3.90**	**0.04**		
	No	79	89				
Resuscitation	Normal Newborn Care	86	92	1.84	0.18		
	Advanced Interventions	14	8				
Congenital Anomalies	Yes	17	4	**8.99**	**0.003**		
	No	83	96				
Type of Anomaly	Cardiac	10	1				
	Hypospadias	1	0				
	Spina Bifida	3	0				
	Tongue tie	1	0				
	Cryptorchidism	1	1				
	Hydronephrosis	1	0				
	Cleft Palate	0	1				
	Accessory Nipple	0	1				

*Note:* Bold values indicate statistically significant.

CI: Confidence Interval.

*t*: Independent *t*‐test.

X^2^: Chi‐square.

## 4 Discussion

This study found several adverse neonatal outcomes associated with GDM. The gestational age and the glycemic level of neonates of pregnant mothers with GDM were significantly lower than those of neonates of mothers without GDM. Neonates of pregnant mothers with GDM required CS for delivery more than neonates of mothers without GDM. Neonates of pregnant mothers with GDM developed RDS and congenital anomalies more than neonates of mothers without GDM. The most common type of congenital anomaly in the neonates of mothers with GDM was cardiac, followed by spina bifida.

This study indicated that the gestational age of neonates of pregnant mothers without GDM was significantly higher than that of neonates of mothers with GDM. This is consistent with previous research that found women with GDM deliver earlier than women without [18]. This is attributed to the fact that GDM can cause many adverse outcomes that lead to early delivery, such as macrosomia and preeclampsia [19, 20]. Our study also found that the glycemic level of neonates of women with GDM is lower than that of neonates of women without. This finding comes in accordance with previous research published by [21]. This could be attributed to increased insulin production by the neonate, sudden cessation of maternal glucose supply to the neonate after birth, and potential delays in the baby's ability to regulate glucose, all of which can contribute to the lower glycemic level, suggesting the need for close monitoring of blood sugar levels in these newborns after birth [22].

The current study found that the risk of CS delivery among women with GDM was significantly higher than that of women without. This finding aligns with many previous studies [14, 23, 24, 25, 26, 27, 28]. It is apparent that many obstetricians prioritize CS as a preferred option due to varied maternal and fetal complications associated with GDM [28]. The main reason for performing CS could be fetal macrosomia; however, CS can also help prevent many adverse obstetric outcomes and serve as a life‐saving measure for both the mother and baby [27].

The current study findings indicate that neonates born to mothers with GDM are at higher risk of developing RDS compared to those born to mothers with no GDM. This comes in agreement with previous research [29, 30]. Scientifically, the risk of neonatal RDS is mainly due to delayed lung maturity, insufficient surfactant production, fetal hyperinsulinemia, and the possibility of preterm or early delivery, all of which are common in diabetic patients owing to increased levels of insulin and insulin‐like growth factors. These factors can cause difficulties in the baby's ability to breathe independently, leading to RDS [29, 30, 31].

This study found that neonates of mothers with GDM are more liable to develop congenital anomalies than neonates of mothers without GDM. The most common type of congenital anomaly was cardiac. This is consistent with the findings reported by previous research that found that GDM is associated with the development of several neonatal congenital anomalies: cyanotic congenital heart disease, hypospadias, cleft lip, cleft palate, gastroschisis, Down syndrome, spina bifida, chromosomal disorder, congenital diaphragmatic hernia, limb reduction defect, anencephaly, and omphalocele [32]. However, a retrospective study showed no significant association between GDM and congenital anomalies except cyanotic congenital heart disease, suggesting the need for more research to establish the relationship between GDM and congenital anomalies of neonates [33].

The current study showed no significant correlation between GDM and neonatal birth weight, specifically macrosomia. However, several studies have reported a significant association between GDM and neonatal macrosomia [19, 34, 35, 36]. The absence of significant relationships between GDM and macrosomia can be attributed to the effectiveness of clinical management strategies employed in the hospital and antenatal clinic for these cases. Also, this study found no significant difference in the hemoglobin A1c between mothers with GDM and those without and again this indicates good treatment strategies and proper patient compliance. These strategies, including dietary management, insulin therapy, and close monitoring of blood glucose levels, may play a vital role in preventing excessive fetal growth even when GDM is present.

In Saudi Arabia, one study from Madina City reported that GDM increased the risk of neonatal hypoglycemia, low APGAR score, intense need for induction of labor, and high neonatal birth weight [8]. Another study from Riyadh City found that several neonatal complications have been noted in infants born to mothers with diabetes, such as macrosomia, hypoglycemia, hypocalcemia, hyperbilirubinemia, RDS, and congenital anomalies. It is supposed that poor diabetes management during pregnancy is linked to these conditions. Additionally, congenital malformations are associated with inadequate diabetes control during the first and second trimesters of pregnancy [17]. A more recent retrospective cohort study from Bisha City found that neonates born to mothers with GDM had a higher risk of adverse neonatal outcomes compared to those born to mothers without GDM. The risks of macrosomia and RDS were significantly higher among neonates born to mothers with GDM [5]. Previous studies in Saudi Arabia found that GDM led to some adverse neonatal outcomes. The results of our study found more adverse outcomes associated with GDM. These include that the gestational age and the glycemic level of neonates of pregnant mothers without GDM were significantly higher than those of mothers with GDM. Neonates of pregnant mothers with GDM required a CS for delivery more than neonates of mothers without GDM. Neonates of pregnant mothers with GDM developed RDS and congenital anomalies more than neonates of mothers without GDM. The most common type of congenital anomaly in the neonates of mothers with GDM was cardiac, followed by spina bifida.

This study provides important information for policymakers and healthcare professionals who follow up pregnant women and prepare for the care of neonates. These findings contribute to the existing knowledge about neonates born to mothers with GDM. The results of this study highlight the importance of comprehensive clinical management strategies for women with GDM to minimize adverse neonatal health outcomes. Given the significant increase in CS delivery among women with GDM, healthcare professionals should provide close monitoring and timely intervention during labor to achieve optimal delivery outcomes. Furthermore, the association between GDM and earlier gestational age, as well as increased risk of neonatal RDS, highlights the need for enhanced prenatal care and neonatal monitoring of this population. The increased risk of congenital malformations among neonates born to mothers with GDM emphasizes the importance of early detection and management of maternal hyperglycemia during pregnancy. Overall, these findings underscore the necessity for individualized healthcare interventions for pregnant women diagnosed with GDM to optimize outcomes for both maternal and neonatal health.

Future research should prioritize several areas to enhance our understanding of the correlation between GDM and adverse neonatal outcomes. Investigating the factors contributing to the increased risk of CS delivery among women with GDM, including both maternal and fetal influences on the mode of delivery, is crucial. Moreover, clarifying the absence of a significant correlation between GDM and neonatal birth weight and APGAR scores requires studies involving larger and more diverse study samples. Furthermore, studies of the mechanisms underlying the increased risk of congenital malformations among neonates born to mothers with GDM are necessary, emphasizing the identification of modifiable risk factors and the development of targeted interventions to enhance neonatal health outcomes. Additionally, future research should consider the impact of continuous monitoring of maternal hyperglycemia levels and compliance with treatment regimens, aiming to optimize clinical management strategies for GDM and improve maternal and neonatal health outcomes.

This study provides valuable insights into the association between GDM and neonatal health outcomes in Jeddah City, employing a retrospective cohort design that directly compared two equal groups of neonates (100 born to mothers with GDM and 100 born to mothers without GDM), allowing for a robust and balanced assessment of the specific impact of GDM on neonatal outcomes. However, several limitations should be considered. Firstly, the study sample was limited to 200 pregnant women who delivered live newborns. A larger, multi‐center study would improve the generalizability of the study. Additionally, the comparison of socio‐demographic characteristics and neonatal health outcomes between the two groups was based exclusively on existing data sets, without the ability to control for potential confounding variables. Future studies should focus on prospective cohort designs to establish stronger causal links, multivariate analysis, adjusting for confounding factors like maternal obesity, gestational weight gain, and socioeconomic status of pregnant women, and long‐term neonatal outcomes beyond the immediate postpartum period.

## 5 Conclusion

In conclusion, this study highlighted significant differences in neonatal outcomes between pregnant women with and without GDM. The gestational age and the glycemic level of neonates of pregnant mothers with GDM were lower than those of neonates of mothers without GDM. Neonates born to mothers with GDM exhibited a higher incidence of CS deliveries, increased rates of RDS, and congenital anomalies compared to those born to mothers without GDM. These findings underscore the importance of understanding the impact of GDM on neonatal health and call for further research to explore the underlying mechanisms and potential interventions to minimize the adverse outcomes. Future studies should prioritize investigating factors contributing to increased risks of CS delivery, RDS, and congenital malformations among neonates born to mothers with GDM, with a focus on developing targeted interventions to enhance neonatal health outcomes and optimize clinical management strategies for GDM. This study assessed how GDM affects neonatal health in Saudi Arabia and provided insights for policymakers and healthcare providers. It emphasized the importance of individualized clinical management for GDM to improve maternal and neonatal outcomes. Future research should focus on understanding factors contributing to adverse outcomes and exploring mechanisms underlying these associations.

## Author Contributions

**Ahmad Ismail:** conceptualization, methodology, software, data curation, investigation, validation, formal analysis, supervision, visualization, project administration, resources, writing – original draft, and writing – review and editing. **Elham Mohammed Ahmed Ezzi:** conceptualization, methodology, data curation, investigation, visualization, project administration, resources, writing – original draft, and writing – review and editing. **Ali Ahmad Al‐Shraifeen:** conceptualization, methodology, data curation, investigation, validation, supervision, visualization, writing – original draft, and writing – review and editing. **Ahmed Ragab:** conceptualization, methodology, investigation, validation, supervision, visualization, writing – original draft, and writing – review and editing. **Mohammad Othman:** conceptualization, methodology, investigation, validation, supervision, visualization, resources, writing – original draft, and writing – review and editing.

## Conflicts of Interest

The authors declare no conflicts of interest.

## Transparency Statement

The lead author Ahmad Ismail affirms that this manuscript is an honest, accurate, and transparent account of the study being reported; that no important aspects of the study have been omitted; and that any discrepancies from the study as planned (and, if relevant, registered) have been explained.

## Data Availability

Coded data and all materials are available upon reasonable request through the author.
